# Surface Interactions between Gold Nanoparticles and Biochar

**DOI:** 10.1038/s41598-017-03916-1

**Published:** 2017-07-10

**Authors:** Minori Uchimiya, Joseph J. Pignatello, Jason C. White, Szu-Lung Hu, Paulo J. Ferreira

**Affiliations:** 1 USDA-ARS Southern Regional Research Center, 1100 Robert E. Lee Boulevard, New Orleans, Louisiana 70124 USA; 2 Department of Environmental Sciences, The Connecticut Agricultural Experiment Station, New Haven, Connecticut 06504 USA; 3 Department of Analytical Chemistry, The Connecticut Agricultural Experiment Station, New Haven, Connecticut 06504 USA; 4 Materials Science and Engineering Program, The University of Texas at Austin, Austin, Texas 78712 USA

## Abstract

Engineered nanomaterials are directly applied to the agricultural soils as a part of pesticide/fertilize formulations or sludge/manure amendments. No prior reports are available to understand the surface interactions between gold nanoparticles (nAu) and soil components, including the charcoal black carbon (biochar). Retention of citrate-capped nAu on 300–700 °C pecan shell biochars occurred rapidly and irreversibly even at neutral pH where retention was less favorable. Uniform organic (primarily citrate ligands) layer on nAu was observable by TEM, and was preserved after the retention by biochar, which resulted in the aggregation or alignment along the edges of multisheets composing biochar. Retention of nAu was (i) greater on biochars than a sandy loam soil, (ii) greater at higher ionic strength and lower pH, and (iii) pyrolysis temperature-dependent: 500 < 700 ≪ 300 °C at pH 3. Collectively, carboxyl-enriched 300 °C biochar likely formed strong hydrogen bonds with the citrate layer of nAu. The charge transfer between the conduction band of nAu and π* continuum of polyaromatic sheets is likely to dominate on 700 °C biochar. Surface area-normalized retention of nAu on biochars was several orders of magnitude higher than negatively charged hydroxyl-bearing environmental surfaces, indicating the importance of black carbon in the environmental fate of engineered nanomaterials.

## Introduction

Engineered nanomaterials are increasingly becoming available as the active ingredients or additives for the direct application on agricultural soils. Examples of commercially available fertilizers and pesticides claiming to contain engineered nanomaterials include Primo Maxx (plant growth regulator by Syngenta; Basel, Switzerland), Nano-Gro (Agro Nanotechnology; Massapequa Park, NJ), nano-Ag answer (PKN fertilizer by Urth Agriculture; Monterey, CA), and nano-5 organic fertilizer (Uno Fortune; Taiwan). Additional routes of entry to agricultural soils include the land-application of sludge^1, 2^ and manure^3^ containing engineered nanomaterials. Despite the increasing agricultural use of nanomaterials, no prior reports are available to understand the extent and reversibility of gold nanoparticles (nAu) retention by soil amendments including biochars. Yet, the current global market size of nAu is estimated to be worth 1.34 billion U.S. dollars, and is projected to increase to 8 billion by 2022^4^.

The Turkevich method^5^ of nAu synthesis employs the reduction of tetrachloroauric acid (HAu^III^Cl_4_) by Na citrate to produce Au^I^ and acetoacetate anions^6^. Citrate capping agents on nAu are fully deprotonated, making nAu negatively charged, even at pH 0–3 where citric acid and dihydrogen citrate dominate the solution-phase speciation^7^. There is an active debate in the literature regarding how tightly citrate is bound to the nAu surface, although no TEM image is available to show the citrate layer on nAu. Park and Shumaker-Parry proposed^8^ that citrate is bound on nAu surfaces through inner-sphere coordination of the central carboxyl group; the terminal carboxyl groups of the bound citrate engage in dangling H-bonds with adjacent bound citrate. Because of this strong H-bonds between the terminal carboxyl groups of the citrate capping agents and associated steric effects, citrate resists release during the thiol functionalization^9^. That is, steric and chelating effects involving the entire layer of H-bonded citrate molecules cannot be fully overcome during the thiol exchange^9^. As a result, citrate remains bound on the facets of nAu and coexists with thiols, rather than engaging in a facile ligand exchange^9^. This indicates a remarkable stability of H-bonding networks involving citrate; the gold-thiolate covalent bond has a strength equivalent to the gold-gold bond^10^. Similar resistance of citrate towards ligand exchange is possible in the presence of environmental dissolved organic carbon (DOC). Dispersion of nAu in the presence of Suwannee River humic acid (SRHA) has been attributed to the negative charge^11^ and steric stabilization provided by the sorbed SRHA^12^.

Biochar is a biomass-derived charcoal (bio-carbon), which has been intentionally added to the agricultural soils worldwide for intended purposes ranging from soil fertilization to carbon sequestration^13^. Biochar releases DOC of varying molecular weight (MW), aromaticity, and carboxyl content, including low MW organic acids structurally similar to citrate^14–16^. Biochar could stabilize nAu by (i) replacing citrate capping agent by its DOC having higher MW and aromaticity, (ii) increasing the solution pH, (iii) providing negatively charged surfaces at pH above its point of zero charge (PZC, typically below 3)^17^, and (iv) serving as a reductant^18, 19^. Conversely, biochar could destabilize nAu by (i) providing attractive surface interactions, (ii) decreasing the solution pH, and (iii) increasing the ionic strength by releasing salts^15^.

Environmental surfaces can destabilize nAu by inducing reversible aggregation and deposition, or irreversible aggregation and precipitation that could involve structural changes in the nAu colloids. The surface interaction of nAu with soil components is not well understood, partly because of the difficulty in distinguishing between the homo-aggregation, hetero-aggregation, and surface binding mechanisms. Retention of nAu was minimal on silica, granite, and other hydroxyl-bearing rock surfaces, unless functionalized by positively charged amines. On the negatively charged bare silica surfaces, 15 nm citrate-nAu underwent heteroaggregation, while 45 nm nAu (produced by borohydride reduction without citrate) did not^20^. Contradictorily, another study reported no interfacial adsorption of citrate-nAu on a negatively-charged silica surface regardless of the particle size^21^. Heteroaggregation of nAu with carbon nanotubes (CNTs)^22^, hematite nanoparticles^23^, and glass bead-filled column^21^ had also been studied. However, reversibility of binding has not been fully examined.

Retention of nAu was irreversible on CNTs in both organic solvents^24, 25^ and water^26^. For nAu stabilized by citrate and other labile, non-covalently bound ligands, which allows for a close approach of particles, charge transfer was proposed to be the dominant attractive mechanism adding to van der Waals^27^. Charge transfer could occur between the conduction band of nAu and π* continuum of CNTs to drive the attractive surface interactions^27^. This transfer of electron density from nAu to the surfaces of CNTs resulted in a close packing of nAu on the outer walls of CNTs^27^. Similar charge transfer between polyaromatic surfaces of biochar and nAu could occur in aqueous media, and will destabilize nAu.

The objective of present study was to understand the roles of citrate capping agents on the extent and reversibility of nAu retention by soil components including biochar. For the first time, TEM was used to image the citrate layer on nAu in the presence and absence of biochar. Experimental conditions were systematically altered to test the additional parameters influencing the surface interactions: biochar’s PZC^17^, aromaticity (H:C ratio), surface morphology (by TEM), and surface area (by CO_2_ porosimetry), and biochar-induced changes in pH, ionic strength, and DOC composition^15^. These controllable parameters will allow us to investigate the relative importance of potential mechanisms: charge neutralization, pore penetration, hydrogen bonding, and charge transfer.

## Results and Discussion

### TEM images of nAu without biochar

**Figure 1:**
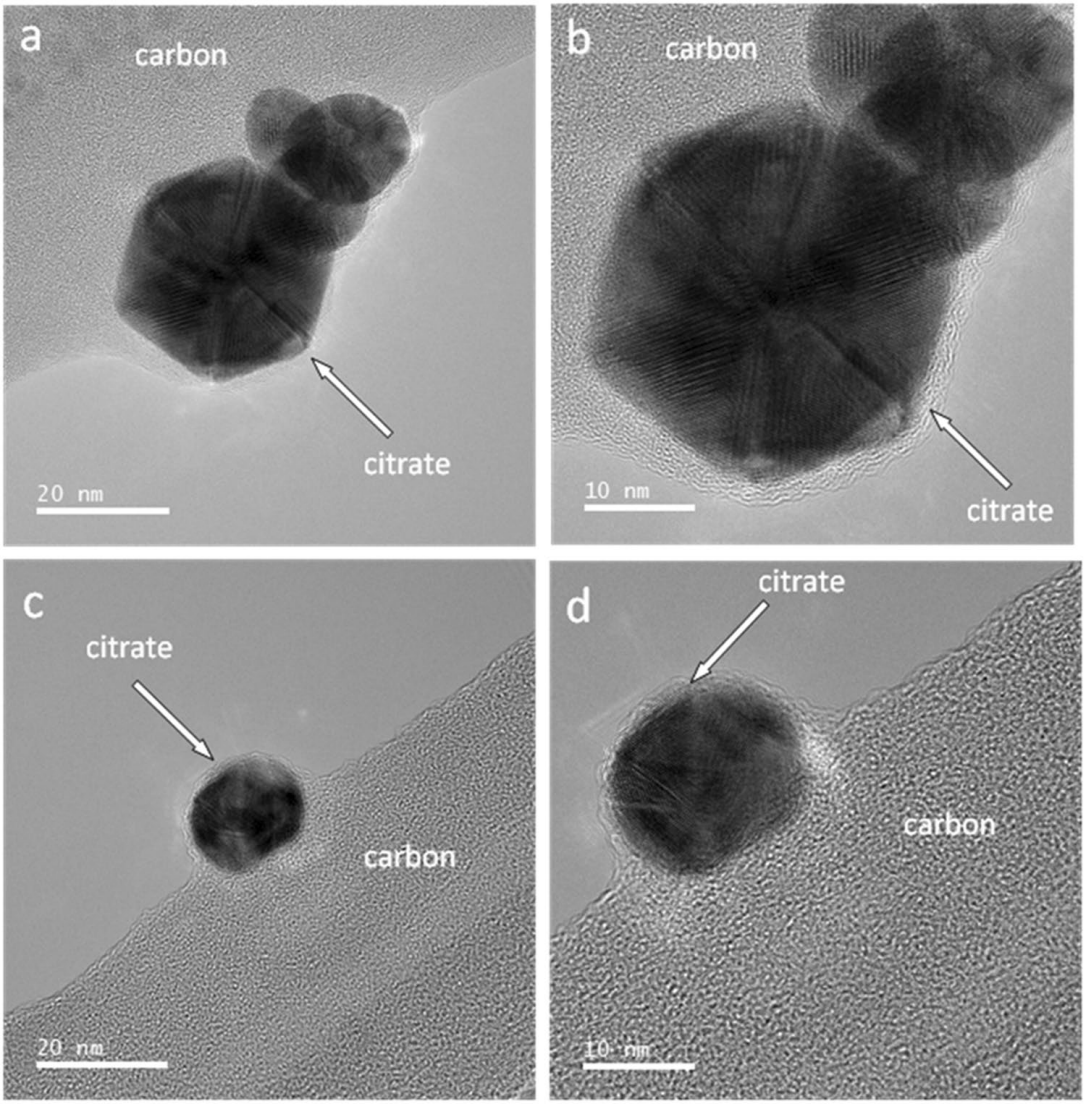
Phase contrast TEM images of individual nAu particles supported on a carbon lacey grid. Images were obtained from two different regions of TEM grid (**a**) and (**c**), each at two different magnifications (**a** and **b**) and (**c** and **d**). The region around the edge of the particles pointed by the white arrows is composed of citrate.

Phase contrast TEM images of nAu particles were obtained from two regions (top and bottom of Fig. 1, each at two different magnifications) of a carbon lacey grid to show representative images. A uniform layer of capping agents (citrate and its decomposition products) is observable on the surface of nAu. Limited reports in the literature utilized TEM to observe the morphology of ligands on nAu surfaces^28^. Uniform coatings of polystyrene ligands caused face-to-face assembly of gold nanocubes, while patchy ligand coatings caused the attachment of same nanocubes at the edges^28^.

### Nanostructures of biochar

A possible nAu retention mechanism is the penetration of biochar’s macropore (>50 nm by the IUPAC definition) having a diameter larger than that of nAu (≈50 nm in Table S2). Grand Canonical Monte Carlo Density Functional theory (GCMC) analysis of CO_2_ isotherm indicated a progressive increase in the surface area of 271–542 m^2^ g^−1^ from 400 to 700 °C biochars (Table S1). Biochars are hereby denoted by the feedstock acronym and pyrolysis temperature, e.g., pecan shell feedstock (PS25) and biochar produced at 700 °C (PS700). Because PS700 showed the highest total surface area (originating primarily from < 2 nm micropores), High Angle Annular Dark Field (HAADF) scanning TEM (STEM) was employed to image the PS700 nanostructure. The mass-thickness contrast technique^29^, including the HAADF STEM, is sensitive to the variations in thickness of a material, and has been used to identify meso- and macropores. As shown in Fig. S1a, ball-milled, 1–2 µm sized PS700 particles had a light gray contrast without visible macropores, while the top left corner of the particle shows a region with brighter contrast. Figure S1b shows a bright-field STEM image of the Ca region (bright region in Fig. S1a). The lattice fringes indicate the crystalline structure composed of Ca, C, and O. Therefore, possible materials are CaO and CaCO_3_. Calculated lattice spacing (0.35 ± 0.02 nm) corresponds to the (100) plane of CaCO_3_. Pecan shell is dense^30^, lignin-rich^31^, and is known to accumulate crystalline Ca oxalate mineral called whewellite (CaC_2_O_4_·H_2_O)^32, 33^. Pyrolysis of Ca oxalate (CaC_2_O_4_) above 400 °C forms Ca carbonate (CaCO_3_), which transforms to calcite (CaO) above 600 °C^34, 35^.

Above observations did not reveal the presence of macropores that will be able to accommodate ≈50 nm nAu. This is in agreement with reported SEM^36^ and microtomography^35^ imaging of pecan shell biochars, as well as a detailed TEM analysis of a model charcoal^37^. In conclusion, there is no visual evidence for the macropores in biochars to accommodate the pore penetration by nAu, although theoretical calculation methods are available to estimate the macropore volume based on the helium or mercury isotherms^38^. Retention of nAu on CaCO_3_ is expected to be minimal because of the charge repulsion^39^. Subsequent sections will focus on the remaining possible interaction mechanisms between nAu and biochars: charge neutralization, hydrogen bonding, and charge transfer.

### nAu retention on biochars and soils

**Figure 2:**
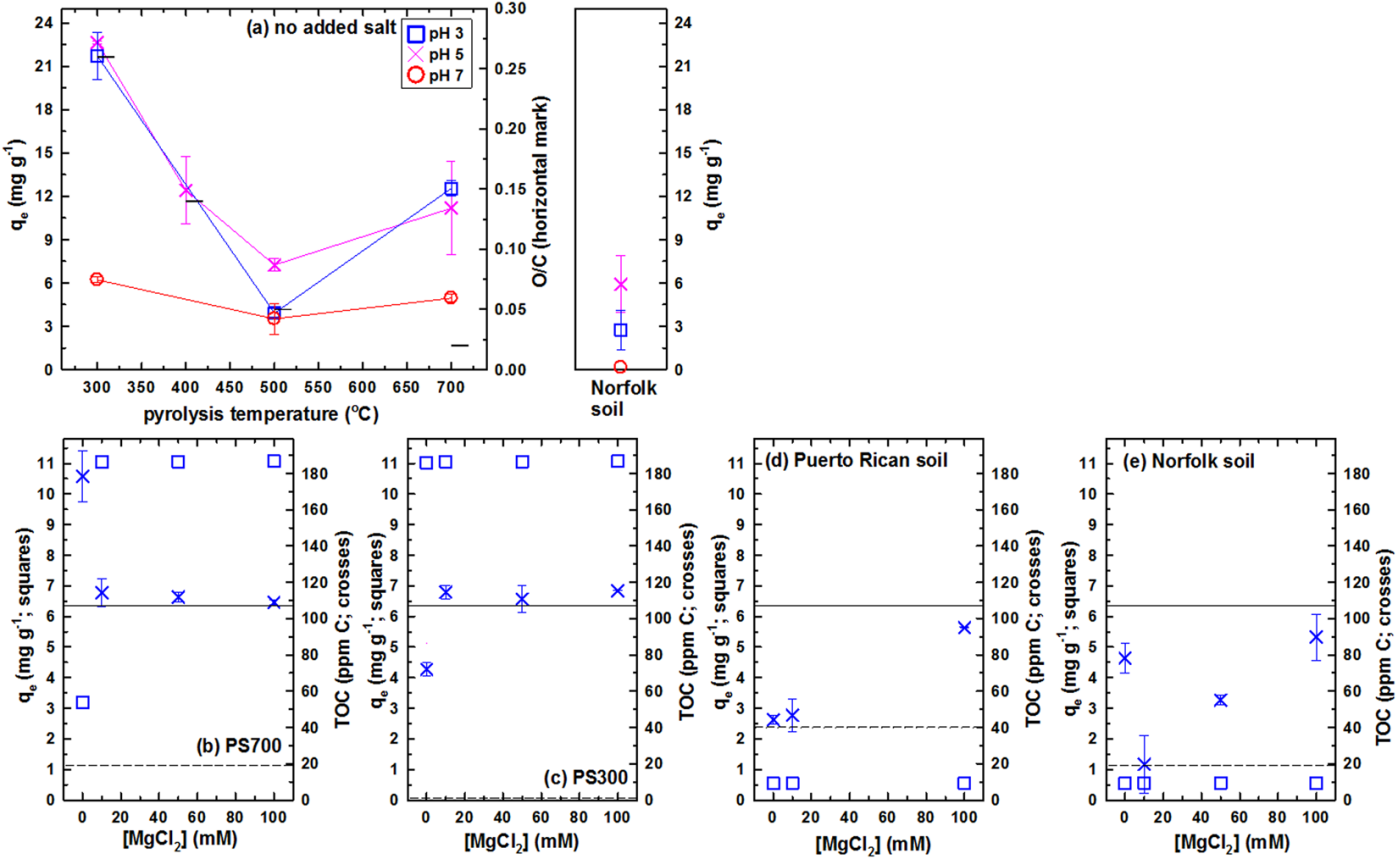
(**a**) Influence of pH on 22 mg L^−1^ nAu (citric acid, pH 3) retention by 300–700 °C biochars and Norfolk soil (1 g L^−1^, 3 d equilibration). (**b**–**e**) Influence of added (10–100 mM) MgCl_2_ on q_e_ (squares for left y-axes) and TOC (crosses for right y-axes) for biochars (1 g L^−1^) and soils (20 g L^−1^). Horizontal lines in b–e represent TOC of nAu stock solution (solid line) and biochar/soil (without nAu, dashed line).

Kinetic experiments employing nAu (Na citrate, pH 7) reached an apparent equilibrium within 8 h, regardless of the solid (Fig. S2, Supporting Information). Figure 2a shows the influence of pH (3–7) and pyrolysis temperature (300–700 °C) on the retention of nAu (q_e_ in mg g^−1^) by pecan shell biochars and Norfolk soil (1 g L^−1^). Error bars in Fig. 2 represent mean ± s.d. for duplicate experiments; lines are for visual aid and do not represent a model fit.

At each pH, the following trend in q_e_ was observed (p < 0.05 by post hoc Tukey): PS300 > PS700 > PS500 ≈ Norfolk soil at pH 3; PS300 > PS500 ≈ Norfolk soil at pH 5; and PH300 ≈ PS700 > Norfolk soil at pH 7 (Fig. 2a). The PS300 is enriched with oxygen-containing functional groups (highest O/C, horizontal marks for the right y-axis in Fig. 2a), while PS700 has the greatest aromaticity (lowest H/C, Table S1). For the 300 and 500 °C biochars, q_e_ decreased with decreasing O/C; however, a further decrease in O/C of the 700 °C biochar increased the nAu retention. Observed trends suggest the contributions of both oxygen-containing functional groups (O/C) and aromatic carbon (H/C) in the retention of nAu by biochars. For a given biochar, the following pH dependence was observed (p < 0.05 by post hoc Tukey): pH 3 ≈ pH 5 > pH 7 for PS300; and pH 5 > pH 3 ≈ pH 7 for PS500 (no significant pH dependence for PS700). Both nAu and biochars (PZC < 3)^17^ are negatively charged within the pH range of Fig. 2a. Therefore, charge neutralization is not the likely cause of nAu retention by biochars. Charge repulsion is expected to restrict nAu retention to the edges, voids, and other defect sites^39, 40^. For example, positively charged dimethylaminopyridine capping agent restricted the intercalation of nAu into the voids (created by sonication) of montmorillonite in the presence of cationic surfactant^40^. In contrast, nonionic surfactant enabled a homogeneous intercalation of nAu^41^. In a pH 9 granite rock suspension, negatively charged nAu was reportedly retained only at the grain boundaries and other defect sites of the negatively charged Fe-bearing (e.g., biotite) minerals^39^.

A putative explanation for the favorable attraction between like-charged surfaces (nAu and biochars) is a strong hydrogen bonding between carboxyl groups on biochar and those on citrate capping agents of nAu. Carboxyl groups are abundant on biochar and primarily responsible for its negative charge^42, 43^. The carboxyl content of biochar typically peaks at 300–400 °C^44^. Hydrogen bonding between the carboxyl groups of adsorbed citrate species and those of the freely-dissolved citrate were the putative cause of irreversible aggregation of nAu during centrifugation at acidic pH^8^. The pK_a1_ of citrate (3.13 at zero ionic strength;^45^ higher on the nAu surface)^46^ is close to the intrinsic pK_a_ of carboxyl groups on biochar (≈3–5)^44, 47^ enriched in the low temperature biochar (PS300). These groups can form low-barrier hydrogen bonds of the type (-CO_2_···H···O_2_C-)^−^, which are among the strongest hydrogen bonds known between organic moieties. In unbuffered biochar or carbon nanotube suspensions, dissolved carboxylate anion can undergo proton exchange with water, releasing OH^−^, and the resulting neutral species forms a hydrogen bond with a surface -COO^−^ group^47–49^. In the absence of nAu, 40 μM citric acid increased the pH of biochar suspensions (10 g L^−1^ of PS300-PS700) after 48 h equilibration, according to the following relationship: pH_t48_ = 1.46 pH_t0_ – 1.47 (r^2^ = 0.98)^50^.

To investigate the extent of citrate-biochar interactions, citrate sorption experiments were conducted on biochars in the absence of nAu. No citrate sorption was observed after incubating 0.5–1 mM citrate and 10 g L^−1^ PS300-PS700 at pH 3–7 for the duration of the nAu retention experiments. Biochar is also known to release ppm-level of low MW carboxyl ligands including lactate, acetate, propionate, and formate^16^. However, a small fraction^50^ of nAu-associate citrate could form strong hydrogen bonds with the carboxyl surface functional groups of biochar to induce retention. Influence of citrate and other chelating agents on sorption is known to be strongly concentration-dependent, resulting in the S-shaped isotherms indicative of two or more opposing mechanisms^51^. For example, a small amount of sorbed citrate on biochars facilitated Cd^II^ uptake, while higher citrate concentration suppressed Cd^II^ sorption on biochar by forming the solution-phase Cd^II^ complexes^50^. Similarly, a small amount of nAu surface-bound citrate, which is visible by TEM (Fig. 1), could form strong hydrogen bonds with the carboxyl functionality of biochars.

The relative proportions of surface-associate and solution-phase citrate in the nAu stock solution are unknown, although centrifugation is used to remove nearly all citrate with a high (≈80%) recovery of citrate-free nAu^52^. The fate of the citrate capping agent was investigated by measuring TOC (crosses for the right y-axes in Fig. 2b–e) of 0.45 μm-filtered supernatant after 3 d reaction between nAu and biochars at pH 3. If the majority of citrate was bound to nAu when nAu is associated with biochar, TOC would decrease to the level of biochar-only control (dashed horizontal lines). Oppositely, if majority of citrate is physisorbed on nAu or released to solution in the presence of biochar, TOC will stay on the horizontal line (TOC of nAu stock solution). As described in detail in Section VI of Supporting Information, TOC remained at the level of nAu stock solution in the presence of biochar. Therefore, only a small fraction of citrate was bound to nAu or biochar when nAu was retained by biochar.

### Reversibility of nAu retention on biochars

**Figure 3:**
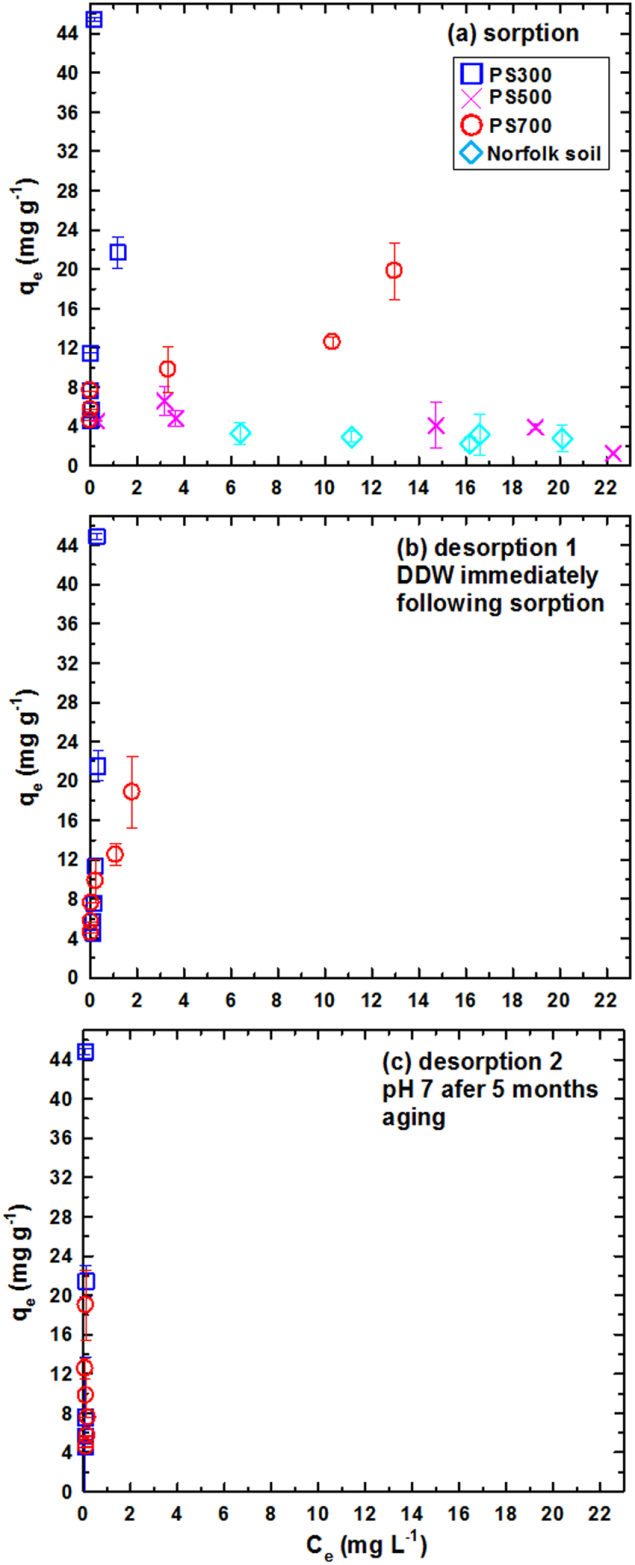
Retention (**a**) and release (**b**–**c**) isotherms of nAu on 300–700 °C biochars and a model sandy loam. Sorption (**a**): 22.9 mg L^−1^ nAu, 0.5–5 g L^−1^ sorbent. Release 1 (**b**): replaced 18 mL supernatant by DDW immediately following (**a**). Release 2 (**c**): replaced 18 mL supernatant by DDW and adjusted pH to 7, after 5 months aging. Each step employed 3 d equilibration and 20 mL total volume.

Greater retention of nAu on biochar relative to a sandy loam (Fig. 2) suggests the importance of attractive charge transfer interaction in addition to van der Waals^27^. The lability of citrate capping agents allows for a close approach of nAu particles to the amorphous carbon surfaces of biochar (Fig. S1), enabling short-range charge transfer interactions^27^. A series of release experiments was undertaken to investigate the reversibility of nAu retention by biochars. Figure 3 presents retention-release isotherms for nAu (citric acid; pH 3, 22 mg L^−1^) on PS300, PS500, PS700, and Norfolk soil (0.5–5 g L^−1^). The pH was buffered by nAu (citric acid) to 2.8 ± 0.1 (mean ± s.d. for 48 experiments) throughout the 3 d equilibration period. Figure 3 reveals the following trend in nAu retention, consistent with the trend in Fig. 2a at pH 3: PS300 ≫ PS700 ≫ PS500 ≈ Norfolk soil. Successive release experiments were conducted for the biochars (PS300 and PS700) that showed significant nAu retention in Fig. 3a. The first release step was initiated by replacing 18 mL out of 20 mL total volume of supernatant by DDW immediately after the retention step. The pH was maintained at 3.3 ± 0.1 (mean ± s.d. for 48 experiments) during the 3-d release period. Release isotherms in Fig. 3b indicate the irreversible retention of nAu on both PS300 and PS700 biochars. Because nAu uptake by the biochars decreased with increasing pH (Fig. 2a), a second release step was conducted at pH 7 to induce the release of nAu. After removing 18 mL supernatant from the first release step (Fig. 3b), the systems were aged for 5 months. The second release step was conducted at pH 7, adjusted using 0.1 M NaOH, for 3 d (final pH was 6.8 ± 0.3). As shown in Fig. 3c, nAu retention was irreversible regardless of pH. As discussed in Section VIII of Supporting Information, fusion of nAu particles could take place during irreversible retention by biochars.

Table S3 of Supporting Information illustrates several orders of magnitude higher number of nAu particles retained per m^2^ surface area of biochar (based on CO_2_ GCMC in Table S1)^53^, compared to a literature value on MWCNTs (based on the BET surface area)^26^. To provide comparison with the charge neutralization mechanism, Table S3 also provides the number of nAu particles imaged per m^2^ area of positively-charged surfaces scanned by AFM or SEM; these values are not directly comparable to biochars normalized to the total surface area of sorbent (calculation methods provided in the Table S3 caption). Overall, Table S3 illustrates the irreversible nAu retention by biochars (1.8 × 10^9^−1.3 × 10^11^ nAu particles per m^2^) exceeding CNTs (3,333 nAu particles per m^2^ at orders of magnitude higher initial nAu concentration, Table S3), even though the charge neutralization and pore penetration mechanisms were determined to be unlikely in the preceding sections.

### TEM imaging of nAu retention by PS700

**Figure 4:**
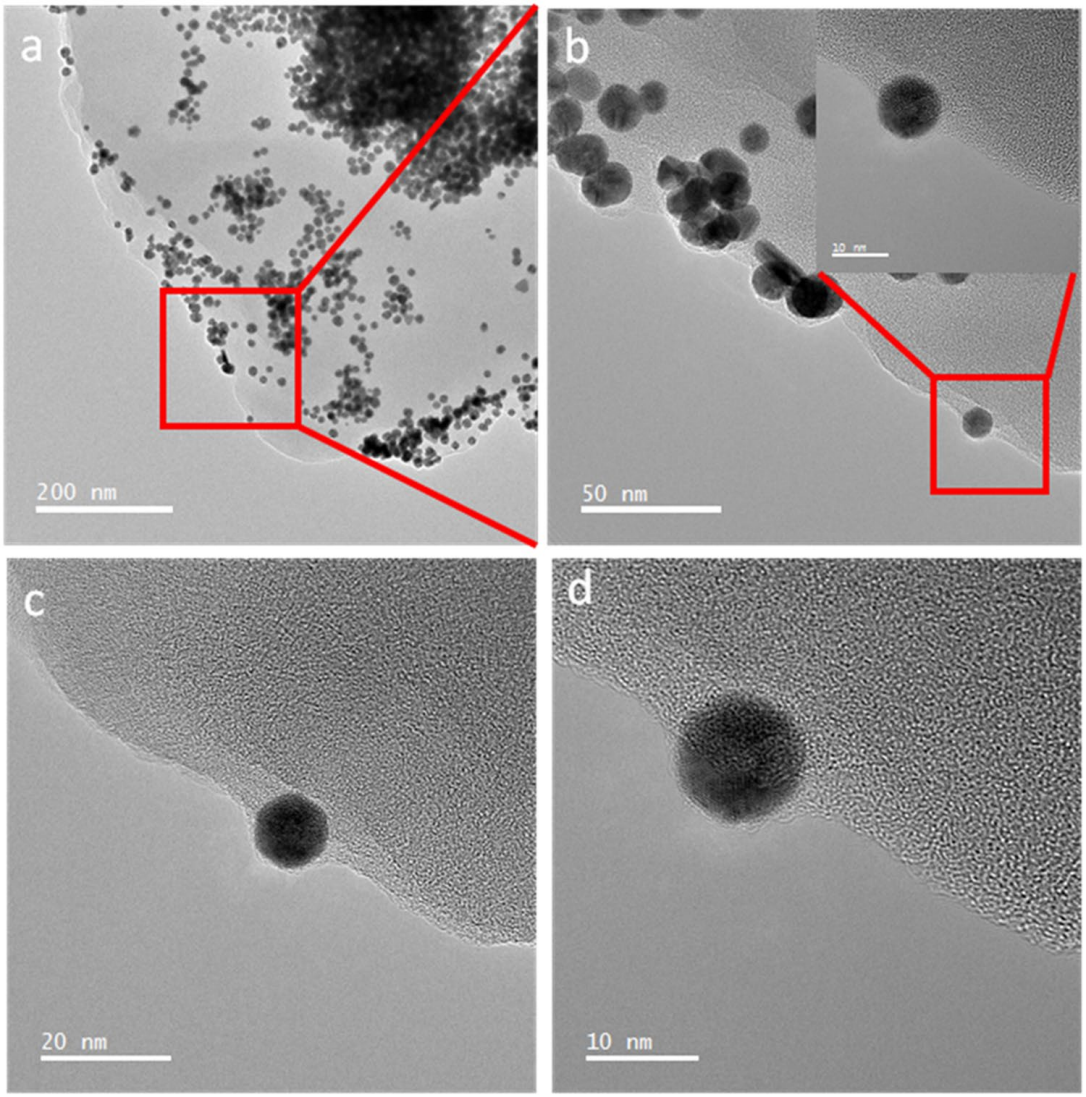
Phase contrast TEM images of nAu + PS700 at different magnifications showing (**a**) nAu aggregates on the biochar multi-sheets and (**b**) alignments along the edges of sheets composing PS700. (**c**) and (**d**) Higher magnification images show the uniform citrate layers preserved on the surface of nAu particles retained by biochar.

Figure 4 shows the phase contrast TEM images of nAu after reaction with biochar (PS700) at four different magnifications. At the lowest magnification (Fig. 4a), nAu particles are either aggregated on the biochar surface (dark region in top right indicating the highest nAu concentration) or aligned on the edges of the sheet layer composing the biochar (left bottom; Fig. 4b shows the higher magnification image of this region). An amorphous layer of citrate, observed in the bare nAu (Fig. 1), is still present on the nAu particles in the presence of biochar (Fig. 4c,d). A multislice computer simulation was performed to demonstrate the influence of defocus conditions on the visibility of amorphous carbon underneath the nAu particles (Section VIII, Supporting Information).

In conclusion, the retention of nAu on biochars was fast and irreversible, and the amorphous and uniform layer of citrate capping agent (which is a small fraction of total citrate present in the nAu stock solution) on nAu was preserved after the retention. Greater nAu retention was observed at lower pH and higher ionic strength. Surface carboxyl as well as the aromatic carbon are likely to be responsible for overcoming the charge repulsion to retain nAu in the following order at pH 3: PS300 > PS700 > PS500 ≈ soil. Extremely high retention (>22 mg g^−1^) of nAu by biochars is remarkable, considering minimal retention of nAu on hydroxyl-bearing granite^39^, sand^54^, clay^40^, and glass^55^ surfaces, unless specifically functionalize to bear the positive charge. Charge transfer and van der Waals involving polyaromatic sheets are likely to be the driving force of attractive interactions, especially for PS700 having the highest aromaticity (lowest H/C in Table S1). Additional attractive forces are provided by the hydrogen bonding between a small amount of nAu-bound citrate capping agent and surface carboxyl enriched in PS300 (highest O/C in Table S1). Vapor deposition of gold on graphene surfaces produced sharp D and D’ Raman bands indicative of the charge transfer between gold and carbon^56^. Similar charge transfer from nAu to amorphous carbon of biochar could be operative for PS700. For lesser condensed but carboxyl-enriched PS300, hydrogen bonding with citrate-nAu is likely to predominate. The lowest, pH-dependent nAu retention ability of PS500 could arise from decarboxylation of biochar above 400 °C pyrolysis temperature^33^.

## Methods

Distilled, deionized water (DDW) with a resistivity of 18 MΩ cm (APS Water Services, Van Nuys, CA) was used in all procedures, and chemical reagents were obtained from Sigma-Aldrich (Milwaukee, WI) at the highest purity available. All glassware was soaked in 20% HNO_3_ overnight, was additionally cleaned with aqua regia, and then triply rinsed with DDW. Total organic carbon (TOC in ppm C) was determined using a Torch combustion TOC/TN analyzer (Teledyne Tekmar, Mason, OH). The pH and electric conductivity were measured using Sartorius Professional PP-15 (Sartorius, Bohemia, NY) and YSI 3200 conductivity (YSI, Yellow Springs, OH) meters. The hydrodynamic diameter of nAu was determined using a Zetasizer Nano ZS-90 (Malvern Instruments, Worcestershire, UK). Production of biochars^50^ and collection of soils^57, 58^ have been reported, and detailed handling and characterization methods are provided in Sections I and III of Supporting Information.

### Gold nanoparticles

Colloidal gold was prepared using sodium citrate or citric acid following the Turkevich method^5^. Briefly, sodium citrate sols were prepared by adding 5 mL of 1% Na citrate solution to a boiling, magnetically-stirred solution of 5 mg gold (III) hydrate (HAuCl_4_·H_2_O) in 95 mL DDW. The solution turned grey-blue and then deep red (no further color change thereafter), and was allowed to boil for additional 1 h, and then cooled to room temperature to obtain the final nAu (Na citrate) stock solution. The resulting nAu (Na citrate) is reported to have spherical shape with the mean diameter of 20 nm^5^. A separate stock solution was prepared by the same procedure using citric acid to produce nAu (citric acid)^5^.

The color, pH, EC (mS cm^−1^), TOC (ppm C), hydrodynamic diameter (nm), Au concentration (mg L^−1^), and λ_max_ (nm) of as-synthesized nAu (Na citrate) and nAu (citric acid) stock solutions are provided in Table S2, Supporting Information. In selected experiments, pH of the nAu stock solution was pre-adjusted using 0.1 M NaOH or HCl. Control experiments indicated that nAu (citric acid; pH 3 as-synthesized) was stable before and after pH adjustment to 5 and 7 for the duration of the experiments described below (3 d to 3 wk). While nAu (Na citrate) was stable as-synthesized for up to 3 wk, pH adjustment to 5 and 3 resulted in a rapid and irreversible aggregation, as reported in the literature^12^. Therefore, only as-synthesized nAu (Na citrate) stock solution (pH 7) was employed in the retention experiments.

### nAu retention-release experiments

Batch experiments were conducted in duplicate using amber glass vials with Teflon lined screw caps (40 mL nominal volume, Thermo Fisher Scientific, Waltham, MA) at 1–10 g biochar L^−1^, and 20–30 mL total volume. Norfolk and Puerto Rican soils (1–20 g soil L^−1^) were employed to provide comparison with biochars. The nAu stock solution was added directly to dry biochar pellets (without dilution or pre-equilibration), and retention isotherms were obtained by varying the biochar loading. In selected experiments, biochar was pre-equilibrated in 10 mM NaCl or MgCl_2_ solutions for 48 h to set ionic strength, and an equal volume of nAu stock solution was subsequently added to initiate the retention experiment. Reactors were equilibrated by shaking end-over-end at 70 rpm for 8 h to 3 wk. Control experiments indicated that nAu was stable at each pH for the duration of the experiment.

At each sampling point, 1 h was allowed for biochar pellets to settle on the bottom of the reactor; centrifugation or other artificial sedimentation procedures were not employed. Subsequently, supernatant was carefully decanted into an empty glass vial, and then filtered (0.45 μm Millipore Millex-GS; Millipore, Billerica, MA). The color of the filtered supernatant ranged from clear, to pink, to red, indicating varying nAu concentrations^26, 59^. Control experiments indicated negligible effects of filtration on the nAu concentration. The nAu in the filtered supernatant was immediately digested in 4 vol% aqua regia. The red/pink-colored solution containing nAu turned clear upon addition of aqua regia, and was allowed to stand overnight. Subsequently, dissolved Au concentration was determined using inductively coupled plasma atomic emission spectrometry (ICP-AES; Profile Plus, Teledyne/Leeman Labs, Hudson, NH). Blanks, blank spikes, and matrix spikes were included for the quality assurance and control for the ICP-AES analysis^60^. One-way ANOVA at a significance level of p < 0.05 was performed using pH and sorbents as the categorical factors, and replications as the random effects by Statistica version 12 (Statsoft, Tulsa, OK). If significant differences existed, post hoc comparison by Tukey’s honest significant difference (HSD) test was performed at a significance level of p < 0.05.

For selected experiments, the release step was initiated immediately following the retention step by replacing 18 mL supernatant (determined gravimetrically; total volume was set to 20 mL in both retention and release steps) by DDW. The suspension was equilibrated for 3 d, and then analyzed by the procedures described above for the retention step. Calculation methods for retention-release isotherms are described in detail in Section II of Supporting Information.

Control experiments were conducted to determine the sorption of citrate on biochars in the absence of nAu over 3 wk period, by following the retention procedure described above. Dissolved citrate concentration in the filtrate was determined using an HPLC system with refractive index detector and Hi-Plex H column (Agilent Technologies, Santa Clara, CA) using 5 mM sulfuric acid mobile phase at 0.6 mL min^−1^ flow rate, and 20 μL injection volume.

### TEM imaging

The nAu stock solution was sonicated in the presence of ethanol for 15 min. One drop of the resulting suspension was deposited on a 200 mesh carbon-lacey Cu grid for TEM observation of individual nAu. Phase contrast TEM images were obtained using a JEOL 2010F TEM (JEOL USA, Peabody, MA), operated at 200 kV. Analogous procedures were following for (i) nAu retained on biochars, and (ii) ball-milled and sieved (400 mesh, <37 µm) 700 °C pecan shell biochar.

## Electronic supplementary material

Supplementary information
