# Supplementary material for: Surface Interactions between Gold Nanoparticles and Biochar
Source: Sci Rep. 2017 Jul 10;7:5027. doi: 10.1038/s41598-017-03916-1 (PMC5503990; doi:10.1038/s41598-017-03916-1)
Supplement: Supplementary file 1 — Supplementary information [file 41598_2017_3916_MOESM1_ESM.doc]

Supporting Information for

Surface Interactions between Gold Nanoparticles and Biochar

Minori Uchimiya^*,a^, Joseph J. Pignatello^b^, Jason C. White^c^, Szu-Lung Hu^d^, Paulo J. Ferreira^d^

*^a^USDA-ARS Southern Regional Research Center, 1100 Robert E. Lee Boulevard, New Orleans, Louisiana 70124, USA*

*^b^Department of Environmental Sciences, The Connecticut Agricultural Experiment Station, New Haven, Connecticut 06504, USA*

*^c^Department of Analytical Chemistry, The Connecticut Agricultural Experiment Station, New Haven, Connecticut 06504, USA*

*^d^Materials Science and Engineering Program, The University of Texas at Austin, Austin, Texas 78712, USA*

*Corresponding author fax: (504) 286-4367, phone: (504) 286-4356, email: sophie.uchimiya@ars.usda.gov

Number of pages: 14

Number of tables: 3

Number of figures: 4

**I. Biochar and soil samples**

**Environmental surfaces: biochars and soils.** Pecan shell feedstock (PS25) was ground (SM 2000 cutting mill, Retsch Gmbh, Haan, Germany) and sieved (<2 mm) prior to pyrolysis at 300, 350, 400, 500, 600, and 700 °C under a flow rate of 1,600 mL min^-1^ N_2_ for 4 h using a laboratory scale box furnace (22 L void volume) with a retort (Lindberg, Type 51662-HR, Watertown, WI). Biochar products were allowed to cool to room temperature overnight under N_2_ atmosphere. Biochars are hereby denoted by the feedstock acronym and pyrolysis temperature, e.g., pecan shell feedstock (PS25) and biochar produced at 350 °C (PS350). Ultimate (C, H, N, S, and O; weight-percent on a moisture- and ash-free basis) and proximate (ash, fixed C, moisture, and volatile matter in weight-percent on a moisture-free basis) analysis results[^1^](#_ENREF_1) for 300-700 °C pecan shell biochars are presented in Table S1, Supporting Information. Table S1 also presents the atomic H/C ratio as an index of aromaticity, and O/C as an index of polarity and hydrophilicity. Grand Canonical Monte Carlo Density Functional theory (GCMC) analysis of CO_2_ isotherm indicated a progressive increase in surface area of 271-542 m^2^ g^-1^ from 400 to 700 °C (Table S1). Low N_2_ BET surface area of biochars has been widely reported,[^2^](#_ENREF_2) and is attributed to the diffusion limitation under the liquid N_2_ temperature.[^3^](#_ENREF_3) The temperature trends in Table S1 are in agreement with the literature.

Norfolk loamy sand was obtained from USDA-ARS Coastal Plains Soil, Water and Plant Research Center (Florence, SC). Collection, handling, and characterization of Norfolk loamy sand had been described.[^6^](#_ENREF_6) Norfolk loamy sand is acidic and eroded, low in TOC, and contains 740, 250, and 10 g kg^-1^ sand, silt, and clay, respectively.[^6^](#_ENREF_6) Puerto Rican humid tropical forest soil was obtained from University of California, Berkeley. As described in detail previously,[^7^](#_ENREF_7) soil samples (0-5 cm depth) were collected at a valley site of a toposequence in Puerto Rico. Puerto Rican valley soil is an acidic (pH 5.01±0.14), clayey (predominantly kaolinite and chlorite), highly weathered and leached Ultisol, containing 9.24% total organic matter and 5.36% TOC.[^7^](#_ENREF_7) All soil samples were air dried and sieved (<2 mm).

**II. Calculation of retention-release isotherms**

The nAu retention isotherms were obtained using the equation traditionally employed to sorption of dissolved pollutant molecules:[^8^](#_ENREF_8)

$$\text{q}_{\text{s}}\text{ = }\frac{\text{V}_{\text{s}}}{\text{m}}\text{ (C}_{\text{i}}\text{-C}_{\text{s}}\text{) (1)}$$

where q_s_ (in mg g^-1^) is the mass of nAu retained on a dry weight basis, C_s_ (in mg L^-1^) is the solution-phase nAu concentration at the end of the retention experiment, C_i_ (mg L^-1^) is the initial nAu concentration (Table S2), V_s_ is the total volume, and m (g) is the dry weight of solid (biochar or soil).[^9^](#_ENREF_9)

Release isotherms were calculated as the mass of nAu that remained associated with the solids at each release step (q_d_ in mg g^-1^) using the following equation:[^10^](#_ENREF_10)

$$\text{q}_{\text{d}}\text{ = }\frac{\text{V}_{\text{d}}}{\text{m}}\text{ (C}_{\text{d-1}}\left(\frac{\text{V}_{\text{d-1,r}}}{\text{V}_{\text{d}}}\right)\text{-C}_{\text{d}}\text{) + q}_{\text{d-1}}\text{ (2)}$$

where V_d-1,r_ is the remaining residual supernatant volume after the removal of supernatant in the (*d*-1)th release step (2 mL determined gravimetrically), V_d_ is the sum of volume added for the *d*th release step and V_d-1,r_, and C_d_ is the equilibrium solution-phase nAu concentration for *d*th release step.

**III. Characterization of biochar and nAu**

**Biochar characterization.** Ultimate (C, H, N, S, and O; weight-percent on a moisture- and ash-free basis) and proximate (ash, fixed C, moisture, and volatile matter in weight-percent on a moisture-free basis) analysis results for 300-700 °C pecan shell biochars are presented in Table 1. Table 1 also presents atomic H/C ratio as an index of aromaticity, and O/C as an index of polarity and hydrophilicity. The trends with temperature in Table 1 are in agreement with the literature. Grand Canonical Monte Carlo Density Functional theory (GCMC) analysis of CO_2_ isotherm indicated a progressive increase in surface area of 271-542 m^2^ g^-1^ from 400 to 700 °C (Table 1). Low N_2_ BET surface area of biochars has been widely reported,[^2^](#_ENREF_2) and is attributed to the diffusion limitation under the liquid N_2_ temperature.[^3^](#_ENREF_3) Point of zero charge (PZC by electrophoretic mobility) of biochars is typically below 3, regardless of pyrolysis temperature.[^11^](#_ENREF_11) Therefore, both biochar[^11^](#_ENREF_11) and nAu[^12^](#_ENREF_12) are expected to be negatively charged within the pH range (3-7) employed in this study.

**Table S1.** Ultimate^a^ and proximate^b^ analysis results of pecan shell biochars pyrolyzed at 300-700 °C.[^1^](#_ENREF_1) Values are given as mean±s.d. of triplicate analysis. Surface area and porosity were determined by N_2_^c^ and CO_2_^d^ isotherms.

^a^Corrected to moisture- and ash-free. ^b^Corrected to moisture-free. ^c^Based on Brunauer-Emmett-Teller fit of the N_2_ adsorption isotherm at 77 K. ^d^Based on Grand Canonical Monte Carlo Density Functional theory analysis of the CO_2_ isotherm at 273.15 K.

**Table S2.** Properties of gold nanoparticle (nAu) stock solutions.

Mean±s.d. of ^a^two and ^b^five replicate analysis. Theoretical [Au] is 28.98 mg L^-1^ based on the reaction stoichiometry.[^13^](#_ENREF_13) In all experiments, pH was buffered by the nAu stock solution: pH 3 for nAu (citric acid), and pH 7 for nAu (Na citrate). The pH dependent experiments employed nAu (citric acid) stock solution pre-adjusted to pH 5 or 7 using 0.1 M NaOH.

**IV. Additional TEM images**


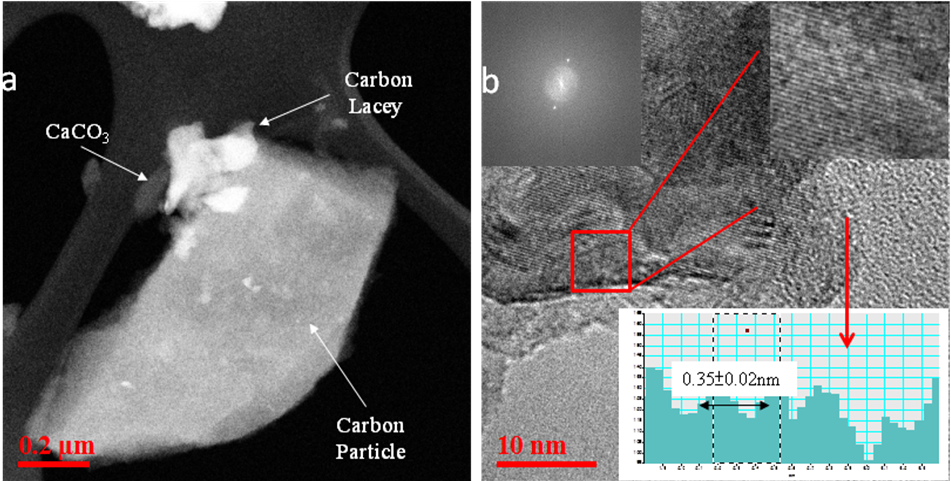


**Figure S1.** (a) High Angle Annular Dark Field (HAADF) STEM image of ball-milled (<37 μm) 700 °C pecan shell biochar on the carbon lacey grid. (b) TEM bright field image of 700 °C pecan shell biochar. The FFT at the top left and the lattice fringes at the top right indicate the presence of crystallinity. Calculated lattice spacing of 0.35±0.02 nm (bottom right) matches the (100) plane of CaCO_3_.

**V. Timecourses of nAu interaction with biochars and soils**

**Figure S2.** Timecourses of nAu sorption on biochars and soils. Initial concentrations were 22.3 mg L^-1^ nAu (Na citrate) in 1 g L^-1^ sorbent. pH was buffered at 6.7 by nAu (Na citrate) stock solution throughout the experiment.

**VI. TOC experiments**

Figures 3b-3e were obtained by: (1) pre-equilibrating biochar (1 g L^-1^) or soil (20 g L^-1^) in 0-100 mM MgCl_2_ for 2 d, and then (2) adding nAu for 3 d equilibration. The initial nAu concentration in Figures 3b-3e (11 mg L^-1^) was lower than in Figure 3a (22 mg L^-1^) because of the pre-equilibration period. As shown in Figure 3b, ≥10 mM MgCl_2_ increased the uptake of nAu by PS700 (q_e_ in mg g^-1^; squares for the left y-axis). Although retention of nAu was nearly complete (i.e., solution-phase concentration of nAu was below the detection limit) in all other experiments presented in Figures 3b-3e, TOC remained close to that in the nAu stock solution (solid horizontal line) in the presence of 300 and 700 °C biochars. Similar results were obtained for 10-100 mM NaCl (instead of MgCl_2_, Figures S1b-S1c, Supporting Information).

The synthesis of nAu employed 2.6 mM citric acid that is equivalent to 187 ppm C (for 147 μM total Au, equivalent to 29 mg L^-1^ total Au).[^13^](#_ENREF_13) Because nAu stock solution was diluted by half after the pre-equilibration period, “theoretical” TOC from nAu stock solution is 94 ppm C. Measured TOC of PS300 (1±0 ppm C without nAu) and PS700 (19±2 ppm C without nAu) are presented as dashed horizontal lines in Figures 5a-5b, and are an order of magnitude lower than the nAu stock solution (measured value of 107±6 ppm C, solid horizontal lines). Therefore, equilibrium TOC (crosses in Figures 5b-5c) originates primarily (>75%)[^14^](#_ENREF_14) from citrate; minor contributions are expected from its oxidation products including acetoacetate,[^15^](#_ENREF_15) acetonedicarboxylate, and acetone that are formed during the nAu synthesis.[^16^](#_ENREF_16)

In Puerto Rican (Figure 3d) and Norfolk (Figure 3e) soils investigated at higher loadings (20 g L^-1^) due to lower nAu retention (Figure 3a), TOC values are consistently below that of nAu stock solution (solid horizontal line). Puerto Rican soil had the highest TOC content (40±1 ppm C in the absence of nAu) of all solids in Figures 3b-3e, and except for 100 mM MgCl_2_, TOC in Figure 3d originates from the soil itself (dashed horizontal line). Puerto Rican soil also had lower equilibrium electrical conductivity (0.2 mS cm^-1^ without nAu) than other solids (0.5-0.6 mS cm^-1^), indicating higher ion exchange capacity originating from clays. A greater amount of citrate was retained by clay-rich Puerto Rican soil than by Norfolk soil (TOC = 19±0 ppm without nAu), except at the highest ionic strength of 100 mM MgCl_2_ (Figures 3d-3e). In conclusion, TOC (originating from mM-level citrate) decreased in the presence of soil (20 g L^-1^), but not biochar up to 10 g L^-1^ with or without nAu.

Rapid aggregation and sedimentation of nAu (Na citrate; pH 5.8, 18.6 nm, -44 mV, λ_max_ = 519 nm) have been reported at the ionic strength above 100 mM and pH below 2.5.[^17^](#_ENREF_17) At a certain threshold pH below the surface pK_a_ of terminal carboxylic acid groups, protonation and neutralization of negative charge decrease the electrostatic repulsion between nAu particles,[^18^](#_ENREF_18) causing irreversible attachment, fusion, and precipitation.[^17^](#_ENREF_17) Intermolecular H-bonds between protonated terminal carboxylates could further drive aggregation by bridging nAu particles.[^19^](#_ENREF_19)

**VII. Influence of NaCl concentration on nAu retention by biochars**

**Figure S3.** Figure 2 b-c replaced by NaCl. Both TOC and q_e_ values as similar to MgCl_2_.

**Table S3.** Extent of nAu retention by biochars (this study) and other surfaces (literature values). Following equation was used to calculate # of nAu particles/m2 biochar surface:[^20^](#_ENREF_20)

$$\begin{matrix} \left(\frac{\text{mg Au}}{\text{g biochar}}\right)\left(\frac{\text{g biochar}}{\text{m}^{2}\text{ by CO}_{2}\text{ GCMC }}\right)\left(\frac{\text{mol Au}}{\text{197 g Au}}\right)\left(\frac{\text{nAu}}{\text{9.01×10}^{\text{-18}}\text{mol Au}}\right) \\ \text{where }\frac{\text{9.01×10}^{\text{-18}}\text{mol Au}}{\text{nAu}}\text{ } \\ \text{= }\frac{\text{π}}{6}\left(\text{density of fcc nAu}\right)\left(\text{diameter in nm}\right)^{3}\left(\frac{1}{\text{atomic weight of Au}}\right)\left(\frac{1}{\text{Avogadro's number}}\right) \\ \text{= }\frac{\text{π}}{6}\left(19.3\frac{\text{g}}{\text{cm}^{3}}\right)\left(\text{56 nm}\right)^{3}\left(\frac{\text{mol Au}}{\text{197 g Au}}\right)\left(\frac{\text{mol Au}}{\text{6.02×10}^{23}\text{Au}}\right) \end{matrix}$$

**VIII. Fusion of nAu**

Fusion of nAu particles is a widely reported mechanism of irreversible aggregation and precipitation. Fusion can be induced by sonication,[^21^](#_ENREF_21) elevated temperature, and sunlight.[^22^](#_ENREF_22) For example, sonication of nAu (Na citrate) formed polydisperse oval- and dumbbell-shaped fused nAu structures.[^22^](#_ENREF_22) Fusion was not observed without sonication, even in the presence of 5 M NaCl that aggregated nAu.[^22^](#_ENREF_22) The synthesis of nAu (Na citrate) itself produces stable fused nanowires.[^23^](#_ENREF_23) Extended (10-15 min) boiling is required to fragment the nanowire intermediates to form the final spherical nAu particles.[^23^](#_ENREF_23) However, fusion is not a prerequisite for the irreversible aggregation of nAu.[^18^](#_ENREF_18) For example, irreversible aggregation (without fusion) of peptide-capped nAu was observed in the presence of oppositely charged citrate bridging ligand in water.[^18^](#_ENREF_18) Similarly, individual nAu particles were irreversibly retained on the outer surfaces of CNTs in chloroform and other organic solvents used to produce composite nAu-CNTs materials.[^24^](#_ENREF_24)

**VIII. TEM computer simulation**


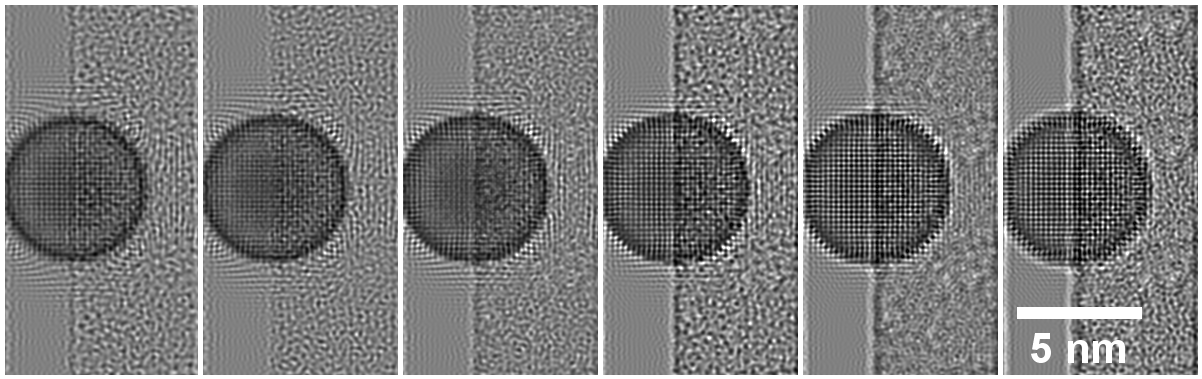


**Figure S4:** Multislice computer simulation of a Au NP on top of amorphous carbon. To simulate a realistic amorphous carbon we have used a tight-binding model (24). The microscope parameters used were an acceleration voltage of 200kV, a spherical aberration coefficient of 1mm and a convergence angle of 0.4 mrad. The images from left to right represent different defocus values, ranging from 0 nm to (-100 nm) with a step size of -20nm.

**References**

(1) Uchimiya, M., Influence of pH, ionic strength, and multidentate ligand on the interaction of Cd^II^ with biochars. *ACS Sustainable Chem. Eng.* **2014,** *2*, 2019-2027.

(2) Lattao, C.; Cao, X.; Mao, J.; Schmidt-Rohr, K.; Pignatello, J. J., Influence of molecular structure and adsorbent properties on sorption of organic compounds to a temperature series of wood chars. *Environ. Sci. Technol.* **2014,** *48*, 4790-4798.

(3) Braida, W. J.; Pignatello, J. J.; Lu, Y. F.; Ravikovitch, P. I.; Neimark, A. V.; Xing, B. S., Sorption hysteresis of benzene in charcoal particles. *Environ. Sci. Technol.* **2003,** *37*, 409-417.

(4) Uchimiya, M.; Wartelle, L. H.; Klasson, K. T.; Fortier, C. A.; Lima, I. M., Influence of pyrolysis temperature on biochar property and function as a heavy metal sorbent in soil. *J. Agr. Food Chem.* **2011,** *59*, 2501–2510.

(5) Shinogi, Y.; Kanri, Y., Pyrolysis of plant, animal and human waste: Physical and chemical characterization of the pyrolytic products *Bioresour. Technol.* **2003,** *90*, 241-247.

(6) Novak, J. M.; Bauer, P. J.; Hunt, P. G., Carbon dynamics under long-term conservation and disk tillage management in a Norfolk loamy sand. *Soil Sci. Soc. Am. J.* **2007,** *71*, 453-456.

(7) Peretyazhko, T.; Sposito, G., Iron(III) reduction and phosphorous solubilization in humid tropical forest soils. *Geochim. Cosmochim. Ac.* **2005,** *69*, 3643-3652.

(8) USEPA OPPTS 835.1220 *Sediment and Soil Adsorption/Desorption Isotherm*; United States Environmental Protection Agency, The Office of Prevention, Pesticides and Toxic Substances, Washington, DC (1998).

(9) Kan, A. T.; Fu, G.; Tomson, M. B., Adsorption/desorption hysteresis in organic pollutant and soil/sediment interaction. *Environ. Sci. Technol.* **1994,** *28*, 859-867.

(10) Limousin, G.; Gaudet, J. P.; Charlet, L.; Szenknect, S.; Barthes, V.; Krimissa, M., Sorption isotherms: A review on physical bases, modeling and measurement. *Appl. Geochem.* **2007,** *22*, 249-275.

(11) Xiao, F.; Pignatello, J. J., Interactions of triazine herbicides with biochar: Steric and electronic effects. *Water Res.* **2015,** *80*, 179-188.

(12) Wright, L. B.; Rodger, P. M.; Walsh, T. R., Structure and properties of citrate overlayers adsorbed at the aqueous Au(111) interface. *Langmuir* **2014,** *30*, 15171-15180.

(13) Turkevich, J.; Stevenson, P. C.; Hillier, J., A study of the nucleation and growth processes in the synthesis of colloidal gold. *Discuss. Faraday Soc.* **1951,** *11*, 55-75.

(14) Balasubramanian, S. K.; Yang, L.; Yung, L. Y. L.; Ong, C. N.; Ong, W. Y.; Yu, L. E., Characterization, purification, and stability of gold nanoparticles. *Biomaterials* **2010,** *31*, 9023-9030.

(15) Park, J. W.; Shumaker-Parry, J. S., Strong resistance of citrate anions on metal nanoparticles to desorption under thiol functionalization. *ACS Nano* **2015,** *9*, 1665-1682.

(16) Zakaria, H. M.; Shah, A.; Konieczny, M.; Hoffmann, J. A.; Nijdam, A. J.; Reeves, M. E., Small molecule- and amino acid-induced aggregation of gold nanoparticles. *Langmuir* **2013,** *29*, 7661-7673.

(17) Diegoli, S.; Manciulea, A. L.; Begum, S.; Jones, I. P.; Lead, J. R.; Preece, J. A., Interaction between manufactured gold nanoparticles and naturally occurring organic macromolecules. *Sci. Total Environ.* **2008,** *402*, 51-61.

(18) Ojea-Jiménez, I.; Puntes, V., Instability of cationic gold nanoparticle bioconjugates: The role of citrate ions. *J. Am. Chem. Soc.* **2009,** *131*, 13320-13327.

(19) Si, S.; Mandal, T. K., pH-controlled reversible assembly of peptide-functionalized gold nanoparticles. *Langmuir* **2007,** *23*, 190-195.

(20) Liu, X.; Atwater, M.; Wang, J.; Huo, Q., Extinction coefficient of gold nanoparticles with different sizes and different capping ligands. *Colloids and Surfaces B: Biointerfaces* **2007,** *58*, 3-7.

(21) Radziuk, D.; Grigoriev, D.; Zhang, W.; Su, D.; Möhwald, H.; Shchukin, D., Ultrasound-assisted fusion of preformed gold nanoparticles. *J. Phys. Chem. C* **2010,** *114*, 1835-1843.

(22) Yin, Y.; Yu, S.; Liu, J.; Jiang, G., Thermal and photoinduced reduction of ionic Au(III) to elemental Au nanoparticles by dissolved organic matter in water: Possible source of naturally occurring Au nanoparticles. *Environ. Sci. Technol.* **2014,** *48*, 2671-2679.

(23) Pong, B. K.; Elim, H. I.; Chong, J. X.; Ji, W.; Trout, B. L.; Lee, J. Y., New insights on the nanoparticle growth mechanism in the citrate reduction of gold(III) salt: Formation of the Au nanowire intermediate and its nonlinear optical properties. *J. Phys. Chem. C* **2007,** *111*, 6281-6287.

(24) Rance, G. A.; Marsh, D. H.; Bourne, S. J.; Reade, T. J.; Khlobystov, A. N., Van der waals interactions between nanotubes and nanoparticles for controlled assembly of composite nanostructures. *ACS Nano* **2010,** *4*, 4920-4928.
